# Effect of Corn Steep Liquor (CSL) and Cassava Wastewater (CW) on Chitin and Chitosan Production by *Cunninghamella elegans* and Their Physicochemical Characteristics and Cytotoxicity

**DOI:** 10.3390/molecules19032771

**Published:** 2014-02-28

**Authors:** Lúcia Raquel Ramos Berger, Thayza Christina Montenegro Stamford, Thatiana Montenegro Stamford-Arnaud, Luciana de Oliveira Franco, Aline Elesbão do Nascimento, Horacinna M. de M. Cavalcante, Rui Oliveira Macedo, Galba Maria de Campos-Takaki

**Affiliations:** 1 Post-Graduate Program in Biological Sciences, Federal University of Pernambuco, Recife 50670420, PE, Brazil; E-Mail: quelberger@hotmail.com; 2 Nucleus of Research in Environmental Science and Biotechnology, Catholic University of Pernambuco, Recife 50050590, PE, Brazil; E-Mail: elesbao@unicap.br; 3 Department of Tropical Medicine, Center of Health Sciences, Federal University of Pernambuco, Recife 50670420, PE, Brazil; E-Mails: thayzastamford@yahoo.com.br (T.C.M.S.); thatianaarnaud@hotmail.com (T.M.S.-A.); 4 Center of Biology Sciences, Department of Microbiology, Federal Rural University of Pernambuco, Recife 52171900, PE, Brazil; E-Mail: lucianafranco@terra.com.br; 5 Department of Pharmaceutical Sciences, Federal University of Paraíba-UFPB, João Pessoa 58051-900, PB, Brazil; E-Mails: horacinnammc@yahoo.com.br (H.M.M.C.); ruimacedo@ccs.ufpb.br (R.O.M.)

**Keywords:** *Cunninghamella elegans*, chitin, chitosan, corn steep liquor, cassava, wastewater, characterization, chorioallantoic membrane

## Abstract

Microbiological processes were used for chitin and chitosan production with *Cunninghamella elegans* UCP/WFCC 0542 grown in different concentrations of two agro-industrial wastes, corn steep liquor (CSL) and cassava wastewater (CW) established using a 2^2^ full factorial design. The polysaccharides were extracted by alkali-acid treatment and characterized by infrared spectroscopy, viscosity, thermal analysis, elemental analysis, scanning electron microscopy and X-ray diffraction. The cytotoxicity of chitosan was evaluated for signs of vascular change on the chorioallantoic membrane of chicken eggs. The highest biomass (9.93 g/L) was obtained in trial 3 (5% CW, 8% CSL), the greatest chitin and chitosan yields were 89.39 mg/g and 57.82 mg/g, respectively, and both were obtained in trial 2 (10% CW, 4% CSL). Chitin and chitosan showed a degree of deacetylation of 40.98% and 88.24%, and a crystalline index of 35.80% and 23.82%, respectively, and chitosan showed low molecular weight (LMW 5.2 × 10^3^ Da). Chitin and chitosan can be considered non-irritating, due to the fact they do not promote vascular change. It was demonstrated that CSL and CW are effective renewable agroindustrial alternative substrates for the production of chitin and chitosan.

## 1. Introduction

In recent years, there has been an increasing interest in biopolymers, specially chitin and chitosan, due to the fact they are easy to obtain, their wide applicability and their promising features such as their absence of toxicity, biodegradability, biocompatibility and environmentally friendly nature and their wide range of potential industrial applications [1]. Chitin and chitosan are natural co-polymers, comprised of units of 2-amino-2-desoxy-d-glycopyranose and of 2-acetamide-2-desoxy-d-glycopyranose interconnected by glycosidic β-1,4 bonds in variable proportions. The first type of these units is frequently present in chitosan [2,3]. Chitin is widely distributed in Nature, being the main component of the exoskeleton of crustaceans and insects and it also occurs in nematodes and in the cell walls of yeast and fungi [4,5].

Chitosan is naturally found in the cell wall of fungi, mainly in the *Zygomycetes* [6,7]. Chitosan is formed by the deacetylation of chitin, and the N-acetyl group can undergo several degrees of deacetylation. Chitosan is a cationic and linear polymer, characterized according to its deacetylation level and its molecular weight which may influence its degradability and polysaccharide hydrolysis. According to its deacetylation level and molecular weight, chitosan’s physical and chemical properties such as solubility, pKa and viscosity will vary. It is difficult to obtain highly deacetylated chitosan because upon hydrolysis the degree of degradation of the polymer increases, thus making the purification process more difficult [8,9]. Chitosan with various molecular weights and degrees of deacetylation has been found to have a wide spectrum of applications as chelating agents in wastewater treatment, an antibacterial and antifungal, and as an immobilizing agent for enzymes or for delivering drugs to their target. Consequently chitosan is widely used in the pharmaceutical, food, agriculture, cosmetics and textile industries [10].

Chitin is commercially obtained from the exoskeletons of marine crustaceans, and chitosan by alkaline deacetylation of chitin at high temperatures for long periods of time [11]. However, these traditional isolation methods of these polymers present some drawbacks and limited potential for industrial acceptance such as the seasonal and limited supply of the raw material while the process of demineralization and deproteinization is aggressive and causes changes in the final product, thereby often lowering its quality since this can cause chemical changes. Consequently these end-product biopolymers often have heterogeneous and inconsistent physiochemical properties. As a result, filamentous fungi have been considered an attractive source of chitin and chitosan for industrial applications because their specific products can be manufactured under standardized conditions [2,3,7,10].

Advances in the fermentation technology for producing fungal chitin and chitosan have been considered as an alternative for overcoming the adverse effects of the traditional extraction of these polymers [5,12,13]. In addition to obtaining microbiological chitin and chitosan with homogenous characteristics and more consistent quality, the biopolymer yields may also be optimized by controlling fermentation and processing parameters such as pH, nutrient concentration in the fermentation medium and the length of incubation time [3,14]. The extraction of these biopolymers is simultaneous; independent of seasonal factors; and the final products do not have the protein contamination that can cause allergic reactions in humans [2,4]. The use of industrial wastes as an alternative nutritional source can favor obtaining a byproduct of high value added [6,15,16] and above all, it can decrease the total production costs by 38% to 73% [17].

Corn steep liquor (CSL) is a residue from the corn processing industry that has a large amount of amino acids, vitamins and minerals. This residue can be considered an alternative substrate for producing fungal chitin and chitosan. Manipueira (cassava wastewater (CW)) is a yellowish liquid obtained from cassava during the cassava flour manufacturing process, and is rich in many nutrients such as potassium, nitrogen, magnesium, phosphorus, calcium and sulfur. Currently, CW is discharged into rivers or released on soil without any kind of treatment, thus causing damage to the environment and human health [6].

On the other hand, for the fungal production of chitosan to be commercially viable, further process optimization at the laboratory and engineering levels is required [18]. Therefore, this article sets out a method for optimizing the production of chitin and chitosan by *Cunninghamella elegans* UCP/WFCC 0542 grown by submersed fermentation and uses two agroindustrial wastes, namely corn steep liquor CSL and cassava wastewater CW, which is an economic alternative for providing carbon and nitrogen at low cost. The physicochemical characteristics and cytotoxicity of the synthesized chitin and chitosan are also described. 

## 2. Results and Discussion

### 2.1.Elemental analysis of Cassava Wastewater (CW) and Corn Steep Liquor

The amount of nitrogen, carbon, oxygen and sulfur presented in the composition of cassava and corn steep liquor CSL is shown in Table 1. The CSL constituted the main nitrogen source, and this waste plus the cassava wastewater CW were the carbon source. Both residues also provide other important nutrients for the metabolism of the microorganism.

**Table 1:** Percentage ofnitrogen, carbon, hydrogen, and sulfur present in the composition of cassavaandcorn steep liquor.

Sample	Nitrogen (%)	Carbon (%)	Hydrogen (%)	Sulfur (%)
Corn Steep Liquor (CSL)	6.49	37.77	6.74	0
Cassava wastewater (CW)	2.04	33.35	6.74	0

### 2.2. Influence of the Culture Media on the Biomass, Chitin and Chitosan Production by C. elegans

Table 2 present the values of biomass production, pH, chitin and chitosan yields obtained in each trial as a response for the 2^2^ factorial design using different CW and CSL concentrations. The responses recorded were biomass production, chitin and chitosan yield by *C.elegans.* An estimate of pure experimental error was calculated from four replicates run corresponding to a central point of the complete factorial (trial 5). These results indicated a higher biomass production (9.93 g/L) during 96 h of fermentation of *Cunninghamella elegans* using conditions 3 (5% CW, 8% CSL). This suggests a culture medium with a greater concentration of CSL in relation to cassava wastewater CW concentrations could favor growth by *C. elegans*. CSL consists of amino acids and carbohydrates which influence the growth of Mucoralean fungi [19]. Similar results were reported by Berger *et al.* [6] and Cardoso *et al.* [20] also showed the positive influence in biomass production of the same concentrations of CSL (8%) used in this study. Probably, the considerably higher carbon and nitrogen contents of CSL compared with cassava wastewater CW, as shown in Table 1, favored this result. Other studies also suggest using CSL as a source of carbon and nitrogen in the composition of culture media instead of glucose and yeast extract, for example by using this substrate to produce succinic acid by *Actinobacillus succinogenes* [21]; and to produce a biosurfactant by the yeast *Candida sphaerica* [22].

**Table 2:** Design matrix for the factorial experiments used to study the influence of factors, cassava wastewater (CW) and corn steep liquor (CSL) concentrations, varied symmetrically around the central point according to the 2^2^ levels.

Assays	Cassava wastewater (CW) % (*v/v*)	Corn steep liquor (CSL) % (*v/v*)	pH	Biomass (g/L)	Chitin (mg/g)	Chitosan (mg/g)
1	5.00	4.00	6.98	6.93	69.57	50.12
2	10.0	4.00	6.98	5.67	89.39	57.82
3	5.00	8.00	6.18	9.93	50.09	44.51
4	10.0	8.00	6.20	8.57	60.71	34.47
5	7.50	6.00	6.50	7.16	70.03	47.37
6	7.50	6.00	6.35	8.00	72.40	48.93
7	7.50	6.00	6.23	7.70	76.64	46.00
8	7.50	6.00	6.48	7.31	70.27	45.81

On the other hand, contrary result was observed with condition 2 (10% CW, 4% CSL) which gave the best chitin (89.39 mg/g) and chitosan (57.82 mg/g) yields. Similar result was obtained by Lins *et al.* [19] who obtained the highest chitosan production by *Rhizopus arrhizus* also using 4% of CSL. There was an increase in the pH range from 5.6 (start of fermentation) to 6.98–6.18. A similar result was observed by Cardoso *et al.* [20]. However, the lower pH in Table 2 (6.18 and 6.20) resulted in the higher yields of biomass. Probably this acidic condition is better for the *C. elegans* growth. The influence of the culture media pH in the biomass yield was also observed by Nwe *et al.* [23] and Nwe and Stevens [14] who observed that the slightly acidic pH values are more favorable to the fungal growth.

Therefore a greater CW concentration and a lower CSL concentration favored these response variables. The addition of CSL in culture media improved chitosan production by *Syncephalastrum racemosum,* but at a 2% concentration [15] which is lower than the concentration shown by trial 2, used in this study. Santos *et al.* [16] also showed the positive effect of this agroindustrial waste as a nutritional source for chitosan production by *Cunninghamella elegans* but using a 0.45% concentration of CSL, which is much less than the 4% concentration used in this study. From these results, considering the high nutritional value of corn steep liquor, it can be suggested that the choice of this waste at lower concentrations could be sufficient to increase chitosan production by *Cunninghamella elegans*.

White *et al.* [24] suggest that the composition of fungal and bacterial cell walls can be attained by genetic manipulation or alteration of culture conditions and consequently, these changes could result in a potential for altering cell wall synthesis in fungi to improve chitosan productivity. The positive influence of high and low concentrations of CW and CSL, respectively, in the production of chitin and chitosan. On the other hand, the growth of the fungus was stimulated in the presence of low concentrations of CW and high concentrations of CSL, a source of nitrogen and carbon.

The best results for biomass, chitin and chitosan production obtained in this study (Table 2) are compared with the literature in Table 3. The different results presented in Table 3 for the chitin and chitosan yields of each microorganism prove that the chitosan content of fungi depends on the fungal strains, the age of the mycelia age, the cultivation medium and the growth conditions and chitin and chitosan extraction method used [2,4,24,25].

**Table 3:** Biomass, chitin and chitosan production by *C. elegans* grown on agroindustrial waste compared with results obtained by the literature.

Microorganism	Substrate	Biomass (g L^−1^)	Chitin (mg g^−1^)	Chitosan (mg g^−1^)	Reference
*Cunninghamella elegans*	CSL and CW	9.93	89.39	57.82	This study
*Rhizopus arrhizus*	CSL and CW	8.80	54.38	20.51	[6]
*Rhizopus arrhizus*	Synthetic medium for Mucorales	-	92	13	[8]
*Rhizopus arrhizus*	Modified synthetic medium for Mucorales	-	94	14	[8]
*Rhizopus arrhizus*	CSL and honey	20.6	-	29.3	[26]
*Rhizopus arrhizus*	CSL 4%	13.00	30.40	12.85	[27]
*Mucor circinelloides*	Yam bean	20.70	500	64	[5]
*Cunninghamella elegans*	Yam bean	24.30	440	66	[3]
*Aspergillus niger*	Potato dextrose broth	9.00	-	107	[16]
*Lentinus edodes*	Potato dextrose broth	1.4	-	33	[16]
*Zygosaccharomyces rouxii*	Yeast malt extract broth	4.4	-	36	[16]
*Candida albicans*	Yeast malt extract broth	1.8	-	44	[16]
*Mucor rouxii*	YPD medium	9–14	-	40–80	[28]
*Mucor racemosus*	YPD medium	15.0	-	35.1	[29]
*Cunninghamella elegans*	YPD medium	25.0	-	20.5	[29]
*Gongronella butleri*	Sweet potato pieces and mineral solution and urea	56.3	-	127 (12.7%)	[30]

- Data not shown.

**Figure 1:**
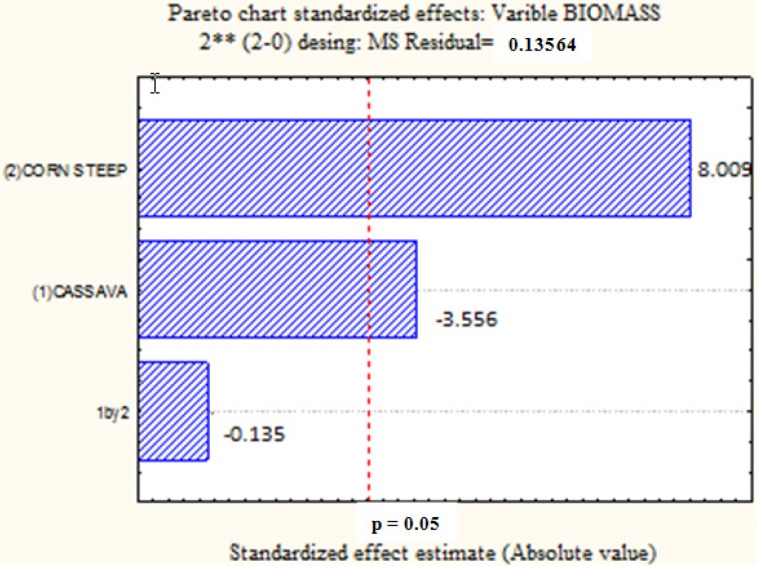
Pareto graph showing the effect of independent variables on the production of biomass by *Cunninghamella elegans* strain UCP/WFCC 0542. Statistically significant (*p* < 0.05).

Figure 1 presents the influence of CW (1), CSL (2) and the interaction between these factors (1 × 2) in the biomass production by *C. elegans,* using a factorial design, with statistical significance (*p* < 0.05). The increase of CSL percentage provided a higher yield of biomass and the opposite was observed when the percentage of CW was increased. The interaction between these factors had a negative effect on biomass production, but not a statistically significant one. These results suggest that, in future research, the biomass production of *C. elegans* could be increased in a culture media with higher CSL and low CW concentrations.

**Figure 2:**
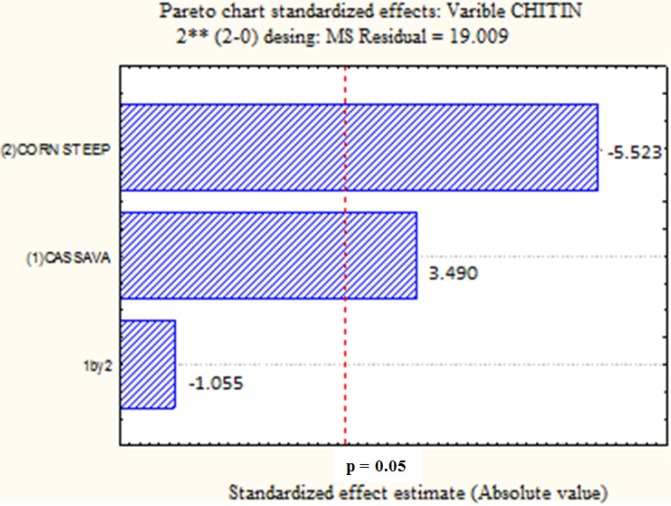
Pareto graph showing the effect of independent variables on the chitin yield by *Cunninghamella elegans* strain UCP/WFCC 0542. Statistically significant (*p* < 0.05).

Figure 2 shows contrary results compared to Figure 1 for the influence of CSL and CW in chitin production. The Pareto chart presents the positive and negative influence of CW and CSL, respectively for chitin production by *Cunninghamella elegans*, with statistical significance (*p* < 0.05)*.* As in Figure 1, the interaction between these two variables did not favor (at least not in a statistically significant way), the production of chitin. These results confirm the data obtained in trial 2 (10% CW, 4% CSL, Table 2).

**Figure 3:**
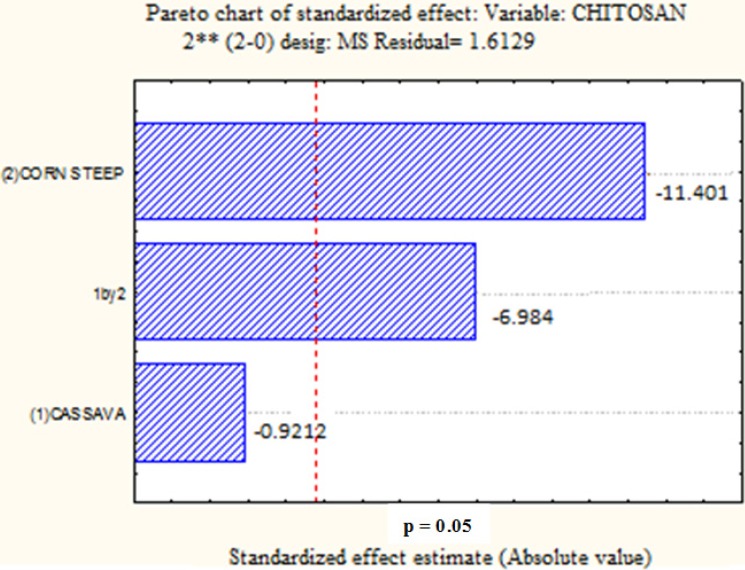
Pareto graph showing the effect of independent variables on the chitosan yield by *Cunninghamella elegans* strain UCP/WFCC 0542. Statistically significant (*p* < 0.05).

Figure 3 presents the negative influence of CW and CSL, and the interaction between these variables, respectively, for chitosan production by *C. elegans.* An increase in the percentage of the two substrates in the culture medium will not favor the production of chitosan by this fungus, which was statistically significant for CSL and interaction of the two wastes (*p* < 0.05) and of no statistical significance for CW (*p* < 0.05)*.* This suggests the use of these wastes in smaller concentrations in the culture medium to achieve better yields of chitosan.

### 2.3. Characterization of Chitosan Extracted from the C. elegans Strain (UCP/WFCC 0542)

#### 2.3.1. Infrared Spectroscopy (Deacetylation Degree—DD%)

**Figure 4:**
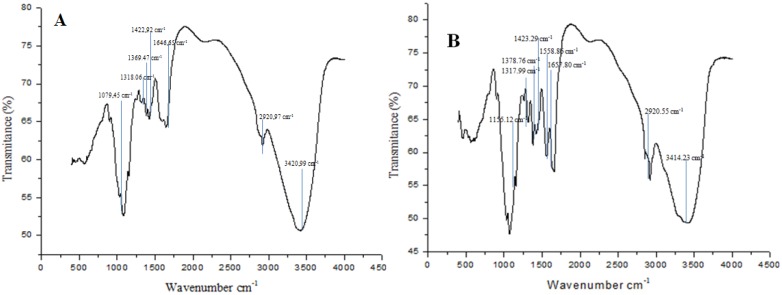
Infrared absorption spectra of microbiological polymers obtained from *Cunninghamella elegans* (**A**) Chitosan; (**B**) Chitin.

The infrared spectra of the biopolymers obtained from *Cunninghamella elegans* are presented in Figure 4. The infrared spectrum of fungal chitin showed the presence of characteristic bands such as 1317.99 cm^−1^, 1558.86 cm^−1^ and 1657.80 cm^−1^, corresponding to CN bond stretching plus CH_3_ wagging; N–H deformation in the CONH plane, including amide II; and carbonyl group stretching, C=O (amide I). In a similar way, chitin shows specific bands at 1155.12 cm^−1^ in the amide II region; 1378.76 cm^−1^, corresponding the C-O stretching of the -CH_2_-OH group (amide II region); 1423.29 cm^−1^, corresponding to the axial deformation of the amide C-N; 2,920.55 cm^−1^, assigned to C-H stretching; and 3414.23 cm^−1^, corresponding to the axial deformation of OH, which appears as overlapping the axial NH deformation band. These results are in agreement with Arbia *et al.* [5]; Chatterjee *et al.* [26]; Ebrahimzadeh *et al.* [27]; Cardoso *et al.* [20]; and Santos *et al.* [28]. The presence of two types of amide group (amide I and II) in the chitin structure is similar to what was observed by Stamford *et al.* [2] and Fai *et al.* [4].

Similar to the infrared spectrum of fungal chitin, the fungal chitosan also presented amide bands as 1318.06 cm^−1^, 1422.92 cm^−1^, 1576.13 cm^−1^ and 1646.65 cm^−1^, but these peaks are less intense than the in fungal chitin, particularly the peak of 1646.65 cm^−1^. Fungal chitosan also presented bands at 2920.97 cm^−1^ and 3420.99 cm^−1^, as observed in the spectrum of fungal chitin. Ebrahimzadeh *et al.* [27] related that during chitosan production, acetyl is eliminated after hydrolysis and, consequently, the carbonyl band gets eliminated in chitosan. However, the infrared spectrum of fungal chitosan with these amide bands, *i.e.*, the presence of an acetyl group on the amino group (stretching, C=O, amide I) shows that the fungal chitosan is not completely deacetylated. The same results were observed by Mario *et al.* [11] who related that, as expected, *N*-deacetylation is associated with a progressive weakening of the band occurring at 1655 cm^−^^1^ (amide I vibrational mode) and the disappearance of the band at 1550 cm^−^^1^ (amide II vibrational mode).

The infrared spectrum of chitin and chitosan is one of the analytical methods used to define the Deacetylation Degree (DD%). The DD% is an important parameter that determines the physicochemical properties of the chitosan because it is linked to the cationic properties of chitosan [4]. The DD% value is directly proportional to the positive charge density on the molecule which confers unique and greater ability to the chitosan for specific industrial, medical or pharmaceutical applications, such as its use as a coagulation agent in physical and chemical waste-treatment systems and as an antimicrobial agent [27,29,30]. The infrared spectra of the chitin and chitosan obtained by *C. elegans* in trial 2 of the 2^2^ factorial experimental design and commercial chitin and chitosan were used to determine the degree of deacetylation. The DD% calculated for fungal chitin and chitosan were 40.98% and 88.24%, respectively. The fungal chitosan value is comparable with the literature, as verified in Table 4.

#### 2.3.2. Viscosity and Molecular Mass

The viscosity of fungal chitosan from *Cunninghamella elegans* (UCP/WFCC 0542) was 3.34 centipoises (cP), considerably lower than the viscosity of crab chitosan with medium (MMW) and mow (LMW) molecular weight (29.7 and 287.2 cP, respectively).

These results were similar to those reported by Pochanavanich and Suntornsuck [10] who stated that the viscosity of fungal chitosan between 3.1 cP to 6.2 cP and commercial crab shell chitosan (Sigma) was 372.7 cP*.* Khalaf [29] obtained a fungal chitosan with 2.7–6.8 cP and crab shell chitosan (Sigma) with 316.2 cP. These results are presented in Table 4.

**Table 4:** Properties of fungal and crab chitosan.

Chitosan sample	DD * (%)	Viscosity (cP)	MW ** (Da)	Reference
*Cunninghamella elegans* UCP/WFCC 0542	82.24 ± 2.0	3.34	5.00 × 10^3^	This study
Crab shell (Sigma)	97.9 ± 0.9	372.7	9.4 × 10^5^	[10]
*Aspergillus niger* TISTR3245	90.9 ± 2.1	6.2	1.4 × 10^5^	[10]
*Rhizopus oryzae* TISTR3189	87.9 ± 2.1	3.5	6.9 × 10^4^	[10]
*Candida albicans* TISTR5239	83.8 ± 0.8	3.1	1.1 × 10^5^	[10]
Crab shell (Sigma)	96.8	316.2	-	[29]
*Aspergillus niger*	84.2	5.9	-	[29]
*Penicillium citrinum*	78.5	4.6	-	[29]
*Fusarium oxysporum*	73.4	2.7	-	[29]
*Rhizopus oryzae*	90.2	6.8	-	[29]
*Penicillium waksamanii*	65.1 ± 3.1	11.3 ± 0.6	-	[29]
*Penicillium citrinum*	62.4 ± 2.7	10.2 ± 0.4	-	[29]
*Penicillium viridicatum*	47.5 ± 1.9	8.9 ± 0.2	-	[29]
*Penicillium aurantiogriseum*	47.3 ± 2.3	9.4 ± 0.2	-	[29]
*Mucor rouxii*	27.3	-	-	[24]
*Rhizopus oryzae*	91.5	7.2	-	[30]
*Aspergillus niger*	89.6	6.4	-	[30]
*Penicillium expansum*	80.2	4.8	-	[30]
*Fusarium moniliforme*	75.3	3.6	-	[30]
Crab shell (Sigma Aldrich)	96.8	316.2	-	[30]
*Gongronella butleri*	92.0	-	3 × 10^4^	[23]
*Mucor rou ii*	82.8–89.8	-	2.48 × 10^4^–5.59 × 10^4^	[26]

- Data not shown, * DD (%) = Degree of deacetylation (%), ** MW = Molecular Weight

The viscosity of the chitosan is directly related with the molecular weight of this biopolymer [30]. High molecular weight chitosan has a higher viscosity than low molecular weight chitosan. Probably, this means that the molecular weight of fungal chitosan may be lower than that of crab chitosan. This was confirmed with the result obtained for the average viscosimetric molecular weight (*M_V_*) of chitosan from *Cunninghamella elegans* obtained in this study, which was 5.2 × 10^3^ g/mol, or low molecular weight. The result is also in agreement with the literature, which reports molar weights ranging between 1.0 × 10^3^ to 9.0 × 10^5^ g/mol [2,6].

Thus fungal chitosan could have potential food, medicinal and agricultural applications as an antimicrobial and preservative agent [10,29]. Omogbai and Ikenebomeh [30] related that the vicosity of chitosan is an important factor which determines its commercial applications and significantly affects its antimicrobial activities. Furthermore, some applications of chitosan are limited by its high molecular weight and viscosity, resulting in low solubility in aqueous solutions such as medical applications which require a low molecular weight chitosan with a high solubility and low viscosity in water at physiologically acceptable pH values [31].

The chitosan of low molecular weight and high DD has a large charge density and a high solubility and is useful in pharmaceuticals, biomedicals and food [23]. In addition, chitosan with a low molecular weight was reported to reduce the tensile strength and elongation of the chitosan membrane, but to increase its permeability. This characteristic can be promising for specific applications [32]. The chitosan with a low viscosity was also reported to have more antimicrobial activity [33]. Ebrahimzadeh *et al.* [27] on relating the results of their study showed that the viscosity of extracted chitosans increased with the increase in DD, but there were some exceptions.

#### 2.3.3. Elementary Analysis

Table 5 shows the carbon, nitrogen and hydrogen percentages of Sigma chitin and chitosan from crustacea, used as standard, and that extracted from *Cunninghamella elegans* under condition 2, which showed the highest yield of these biopolymers.

**Table 5:** Percentage of carbon, nitrogen, hydrogen, carbon/nitrogen and degree of deacetylation of chitin and chitosan samples of crustacean (standard) and extracted from *Cunninghamella elegans*.

Sample	% N	% C	% H	C/N
Crustacean Chitin (Sigma)	5.30	40.25	7.11	7.59
Crustacean Chitosan (Sigma)	7.03	37.88	6.59	5.39
Fungal Chitosan	5.59	30.52	7.66	5.46
Fungal Chitin	5.27	40.69	9.13	7.53

The fungal chitosan 2 showed lower nitrogen (5.59%) and carbon (30.52%) content than the Sigma crustacean chitosan (7.03% of N, 37.88% of C) and the crustacean chitosan (41.2% to 44.5% carbon and 7.0% to 8.5% nitrogen) obtained by Santos *et al.* [16]. However, the C/N ratio of the fungal chitosan (5.46) is similar to the calculated values of C/N obtained by Santos *et al.* [28].

The nitrogen content of chitosan increases with a longer deacetylation reaction and a more efficient deacetylation of chitin with a higher degree of deacetylation of chitosan. This was also observed by Yen *et al.* [34] who treated the crustacean chitin with 30 ml of 40% sodium hydroxide solution at 105 °C for 60, 90 and 120 min to obtain the chitosan denominated: C60, C90 and C120, respectively. Chitosan C120 showed the higher nitrogen content (9.5% ± 0.2%) and degree of deacetylation (93.3% ± 0.4%). Probably, in this study, the degree of deacetylation (82%) and nitrogen content (5.59%) of the fungal chitosan 2 were consequences of the low sodium hydroxide solution concentration (1 M or 4% NaOH) and of the short period (15 min) of deacetylation used, when compared with the methodology by Yen *et al.* [34]. In addition, the carbon content of chitosan is lower than that of chitin due to the loss of acetamido groups during the deacetylation reaction, and the lower the carbon content of chitosan, the higher the degree of deacetylation. A similar result is observed in fungal chitosan 2 with a lower carbon content (25.10%) than fungal chitin 2 (39.69%).

#### 2.3.4. Thermal Analysis

**Figure 5:**
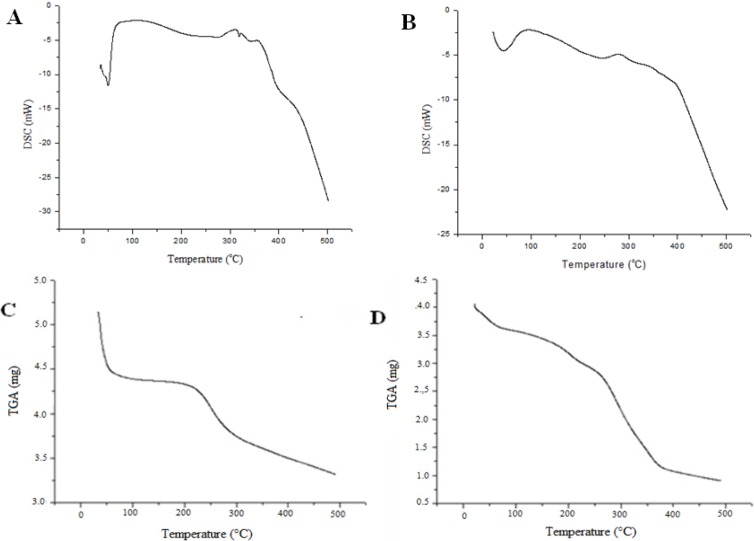
Microbiological biopolymers from *Cunninghamella elegans* UCP/WFCC 0542: DSC curves for chitosan (**A**), chitin (**B**) and TGA thermograms for chitosan (**C**) and chitin (**D**), under continuous flow of dry nitrogen gas (50 mL min^−1^), at a heating rate of (10 °C min^−1^).

The thermal properties of chitin and chitosan samples obtained from TGA–DSC over a temperature range of 0–500 °C are illustrated in Figure 5.

The thermograms were characterized by the peak temperature of endotherms corresponding to water evaporation, which depends on the sample drying process, and the peak temperature of exotherms representing the decomposition of amine units in the polymer as related by Sreenivasan [35] and Santos *et al.* [28]. The first registered thermal event was an endothermic peak at 51.38 °C and 42 °C for chitosan and chitin, respectively; and these peaks are expected to reflect physical and molecular changes during N-deacetylation and carboxymethylation [4]. Each endo- or exothermic peak temperature and area changed as a function of primary and higher order structures of the macromolecule [36]. These biopolymers showed a similar trend in DSC and TGA but the chitin had higher thermal stability than the corresponding chitosan. Consequently, the endothermic peak area was higher for chitosan, 310.25 °C (Figure 5A) than for chitin, 280.57 °C (Figure 5B), i.e., the endothermic peak area increased with the increase in N-deacetylation and carboxymethylation, indicating that a definite correlation exists between the water holding capacity and chemical and supramolecular structure of these polymers, as observed by Zhang *et al.* [37] and Yen and Mau [38]. The DSC was used as an effective technique to correlate the heat of the reaction to the degree of deacetylation (DD%) and carboxymethylation [36].

The two DSC processes are consistent with the trend observed in the TGA curves. The TGA measurements indicate that chitosan and chitin samples lost water at a relatively low temperature (below 100 °C, Figure 5C,D). This means that this water is physically adsorbed and/or weakly hydrogen-bonded to chitosan molecules (stage 1), as shown by Zawadzki and Kaczmarek [39]. The second stage, which began at 172 °C for chitosan and at 109 °C for chitin, is due to the decomposition temperature of these polymers with a carbonized residue formation [4]. Most probably in this stage, the hydrogen-bonded water is released [39]. The third weight loss point was above 300 °C which represents the start of carbonized material consumption, a result similar to that found by Fai *et al.* [4] and Liu *et al.* [40].

#### 2.3.5. Scanning Electron Microscopy

**Figure 6:**
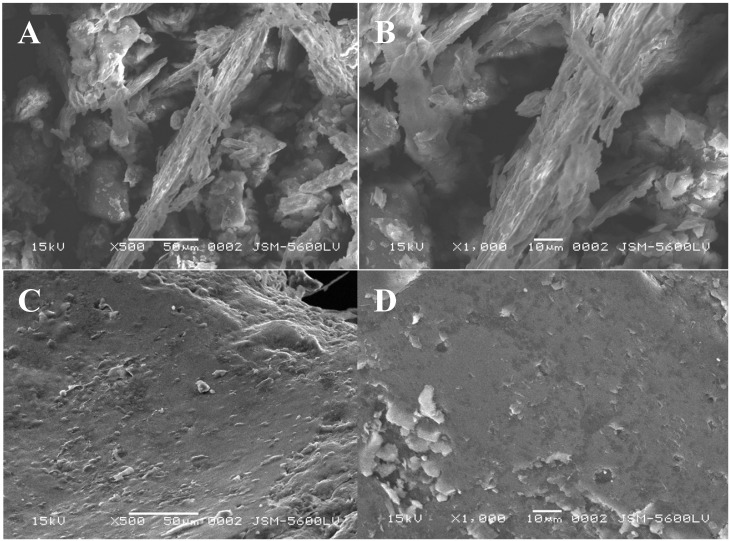
Microbiological biopolymers from *Cunninghamella elegans* UCP/WFCC 0542: SEM electromicrographies of (**A**) chitin at 500× magnification, (**B**) chitin at 1000× magnification, (**C**) chitosan at 500× magnification and (**D**) chitosan at 1000× magnification. The measurement bar = 50 µm.

The chitin and chitosan produced by *Cunninghamella elegans* growth in trial 2 were selected for examination by scanning electron microscopy (SEM, Figure 6). The chitin showed a prominent arranged microfibrillar crystalline structure (Figure 6A,B) which was absent in the chitosan (Figure 6C,D). Similar results were observed in crustacean chitin by Yen *et al.* [34], and Arbia *et al.* [5], and in fungal chitin by Chan, Chen, and Yuan [41]. Also, fungal chitosans did not show the microfibrillar structure in SEM [38]. Yen *et al.* [34] related that the crystalline structure observed between fungal and crab chitins might also be attributed to their different intersheet or intrasheet hydrogen-bonding systems. However, the preview of this microfibrilar structure in the chitin produced by *C. elegans* may have been caused by the process for extracting this biopolymer *i.e.*, the deproteinization step.

The fungal chitosan also exhibited a structure that was more compact and dense than the fungal chitin nor was this chitosan porous. This biopolymer also showed crumbling layers of flakes as observed in crustacean chitosan by Yen *et al.* [34] and few aggregated flakes with a dense and firm structure, without porosity, as related by Yen and Mau [38] for fungal chitosan. These authors compared different chitosans by SEM after a longer N-deacetylation step that used a sodium hydroxide solution at 105 °C, for 60, 90 and 120 min, and they stated that it seems that the longer the deacetylation, the more clearly the cloudy and fibrillar crystalline structure of chitosans was observed. Probably, the same structure could be observed in the fungal chitosan obtained in this study if deacetyation were to be more prolonged. The fungal chitosan obtained in this study also showed rough surfaces, devoid of a recognizable irregular spatial pattern.

#### 2.3.6. X-ray Diffraction

**Figure 7:**
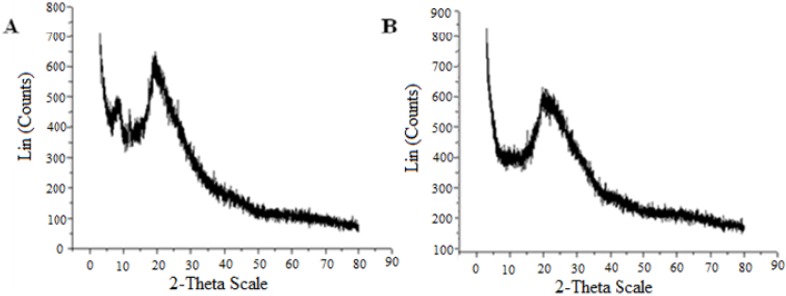
X-ray diffractograms of chitosan (**A**) and chitin (**B**) obtained from *Cunninghamella elegans*.

The specifics in the polymorphic form and crystalline structures of chitin and chitosan were determined by X-ray diffraction. These patterns are indicative of different spacing in the crystal planes, and a polymorphic structure. Diffraction provides accurate measurements of the crystalline content, which greatly affects physical and biological properties of the polymer [4,16,26]. The powder diffraction pattern of chitosan (Figure 7A) from *Cunninghamella elegans* grown in trial 2 (Table 3) showed strong Bragg refractions at an angle of 20.0° 2*θ* and 9.0° which are two characteristic peaks of chitosan, as observed by Wang *et al.* [42]. However, one peak at about 9.00° disappeared in the fungal chitin. Probably a purification process would be necessary to obtain a satisfactory biopolymer, and a similar result was described by Zhang *et al.* [37].

The crystallinity indices of chitin and chitosan grown from *C. elegans* in the best trial 2 of the factorial design and of commercial chitin and chitosan (Sigma) were determined from the scattering intensity at two angles, one at 2*θ =* 9–10° and the other at 2*θ =* 19–20° (Figure 7). The results are supported by the literature [26,37], in which the crystallinity indices of chitin obtained from *Cunninghamella elegans* biomass in trial 2 (35.80%) and of commercial chitin (45.63%) were higher than the chitosan from *C. elegans* biomass in trial 2 (21.17%) and commercial chitosan (23.82%). The higher crystalline index of chitin reflected its higher degree of crystallinity and a more ordered structure [43]. This crystallinity is reinforced by the prominent arranged microfibrillar crystalline structure of chitin in SEM (Figure 6A,B). The lower crystallinity of chitosan indicates disruption of intra- and inter- molecular hydrogen bonds, and the lower crystallinity of fungal chitin and chitosan produced by *C. elegans* might indicate their improved water solubility in comparison with commercial chitin and chitosan, probably due to the more severe extraction conditions during thermochemical extraction, as related by Zhang *et al.* [37]. This lower crystallinity of fungal chitosan in relation commercial chitosan to is supported by the intensity of its peak which was also less than the peak of commercial chitosan around 20°. With these results, and based on the literature, it can be concluded that fungal chitosan displays an organized reticular structure and its crystallinity is related to the DD% function [4,26].

### 2.4. Cytotoxicity Test using Chorioallantoic Membrane (HET-CAM Test)

The cytotoxicity of chitin and chitosan were verified using the HET-CAM test. Cytotoxicity was evaluated for development of irritation symptoms, such as haemorrhage, coagulation (intra- and extravascular protein denaturation) and vasoconstriction, when the test substances were added to the membrane and left in contact for 5 min (Table 6). The polymers were shown to be non-irritating (IS = 0.0) because they did not prompt vasoconstriction, haemorrhage or coagulation in the CAM within 5 min.

**Table 6:** Cytotoxicity of chitin and chitosan evaluated for the development of irritant endpoints: vasoconstriction, haemorrhage and coagulation.

Assays	Chitin	Chitosan	1% SLS
Vasoconstriction	0.0	0.0	6.0 ±1.0
Haemorrhage	0.0	0.0	48 ±3.0
Coagulation	0.0	0.0	63 ±3.0
Irritation potential	0.0	0.0	17.74 ± 0.4

Non-irritating: 0–0.9; slightly irritating: 1–4.9; Irritating: 5–8.9 and severely irritating: 9–21; Positive control: 1% sodium lauryl sulfate (SLS); mean values (%) in quintuplicate.

The CAM has been proposed as a model for a living membrane because it has a functional vasculature. The acute effects induced by a test substance on the small blood vessels and proteins of this soft tissue membrane are proposed to be similar to those of the rabbit eye test, while offering the advantages of being more universally acceptable as it is a non-animal test and is completed more rapidly [44]. Several studies have been conducted to evaluate the feasibility of using HET-CAM as a complete replacement for the *in vivo* rabbit ocular test. This test has several advantages, including due to its being simple, rapid, sensitive, easily performed and relatively cheap [45,46].

In Germany and France, HET-CAM has been officially accepted as a valid *in vitro* assay, at least for predicting several irritating substances [47]. Current laws regulating animal experimentation allow protocols that use chick embryos without authorisation from animal experimentation committees; however, in the UK, the British Animal Welfare Act (1986) states that an embryo egg up to 10 days of gestational age can be used as a non-animal test [44,45].

## 3. Experimental

### 3.1. Microorganism and Maintenance

A *Cunninghamella elegans* strain UCP/WFCC 0542 was isolated from mangrove sediments situated in Rio Formoso, Pernambuco State of Brazil, and belongs to the Culture Collection of the “Universidade Católica de Pernambuco” (UCP), located in the Nucleus of Research in Environmental Sciences, Catholic University of Pernambuco, Brazil —NPCIAMB/UNICAP. The Culture Collection is registered in the World Federation for Culture Collection (WFCC). The fungus is maintained on potato dextrose agar (PDA) medium at 5 °C. The culture is transferred to a new medium every four months.

### 3.2. Chemicals, Cassava Wastewater (CW) and Corn Steep Liquor

All reagents used were of analytical grade. The acetic acid and NaOH were obtained from Vetec (São Paulo, PB, Brazil), and the crustacean chitin and chitosan from Sigma Aldrich (St. Louis, MO, USA). The tropical residue cassava wastewater (CW) was kindly provided from local industry and corn steep liquor (CSL), which is a byproduct of the corn manufacturing industry (kindly donated by Corn Products do Brasil, Cabo de Santo Agostinho, PE, Brazil) was used as the soluble substrate. These agro-industrial wastes were used as the carbon and nitrogen source as per the 2^2^ factorial designs.

### 3.3. Elementary Analysis of Cassava Wastewater (CW) and Corn Steep Liquor

The elementary analysis of CW and CSL was carried out on a model EA 1110 Carlo Erba Instruments (Milan, Italy) elemental analyzer.

### 3.4. Biomass Production by C. elegans

*Cunninghamella elegans* was grown in Petri dishes (9 cm in diameter), containing PDA medium at 28 °C for 8 days. A suspension was prepared and counted to 10^7^ sporangioles/mL, using a hematocytometer. Petri dishes with PDA were inoculated with 1 mL of the sporangiole suspension and maintained for 18 h at 28 °C. At the end of the desired incubation period a total of 20 discs of PDA medium (1 cm diameter) with the mycelium of *C. elegans* were inoculated in Erlenmeyer flasks containing 200 mL of the alternative medium, pH 5.6, with varying levels of corn steep liquor (CSL) and cassava wastewater (CW) concentrations. These parameters were varied symmetrically around the central point according to the 2^2^ factorial design (Table 1 and Table 2). The flasks were incubated at 28 °C in an orbital shaker at 150 rpm, for 24 h. Thereafter, the culture used as pre-inoculum was transferred to the Erlenmeyer flasks with 150 mL of the alternative medium, pH 5.6 and incubated at 28 °C in an orbital shaker at 150 rpm for 72 h. The mycelia were harvested, washed twice in distilled water by filtration, using a nylon membrane silkscreen (120 F), and underwent lyophilization. Afterwards, the biomass was maintained in a vacuum dissector until constant weight.

### 3.5. Factorial Design

A 2^2^ full factorial design was carried out to analyze the main effects and interactions of cassava wastewater (CW) (5%–10%) and corn steep liquor (CSL) (4%–8%) on the response variable of biomass, chitin and chitosan yield by *C. elegans* and to select the best condition for the production of the mycelia and biopolymers as per the variables established (Table 7). The Pareto diagrams were compiled to validate the influence between these agro-industrial wastewaters (independent variables) and the response variables. An estimate of pure experimental error was calculated from four replicates run corresponding to a central point of the complete factorial. The data obtained from the experiments were subjected to statistical analysis by STATISTICA software version 7.0 (StatSoft Inc., Tulsa, OK, USA) and the significance of the results was tested at *p* < 0.05 level.

**Table 7:** Design matrix for the factorial experiments used to evaluate the influence of 2 factors (cassava wastewater (CW) and corn steep liquor) on biomass, chitin and chitosan production by *Cunninghamella elegans* UCP/WFCC 0542, with experimental conditions set at the mean of two extreme levels (−1 and +1) and a central point (0).

Independent Variable		Factor levels	
	−1	0	+1
Cassava wastewater (CW) % (*v/v*)	5.0	7.50	10.00
Corn steep liquor (CSL) % (*v/v*)	4.00	6.00	8.00

### 3.6. Determination of pH

After culture, the pH of the cell-free metabolic liquid was determined by potentiometry. All experiments were performed in triplicate.

### 3.7. Chitin and Chitosan Extraction

The extraction of chitin and chitosan was carried out using dry biomass of *Cunninghamella elegans* following the methodology of Hu *et al.* [48]. After drying the biomass, it was treated with 1 M NaOH solution (1:30 *w/v*, 121 °C, 15 min). Alkali-insoluble material was obtained by centrifugation (4,000 g, 20 °C, 10 min), and extracted using 2% of acetic acid (1:30 *w/v*, 100 °C, 15 min) followed by centrifugation at 4,000 g, 20 °C, 15 min. The supernatant was obtained and the pH was adjusted to 10, and maintained overnight at 5 °C, until the chitosan fraction was precipitated. The chitosan was obtained by centrifugation at 4,000 g, and washed with distilled water four times, freeze-dried, and kept in a dissecator until constant weight.

### 3.8. Characterization of Chitin and Chitosan

#### 3.8.1. Infrared Spectroscopy (Deacetylation Degree–DD%)

The degree of deacetylation (DD%) for microbial chitin and chitosan were determined using infrared spectroscopy as per Baxter *et al.* [49], using the absorbance ratio A1655/A3450 and calculated as shown in Equation (1):

DD (%) = 100 − [(A1655/A3450) × 115]
(1)

Two milligram samples of fungal chitin and chitosan, which had been dried overnight at 60 °C under reduced pressure, were thoroughly blended with 100 mg of KBr, to produce 0.5 mm thick disks. The disks were dried for 24 h at 110 °C under reduced pressure. Infrared spectrometry was undertaken with a Bruker 66 Spectrometer (Bruker Optics, Ettlingen, German), using 100 mg KBr disks for reference.

#### 3.8.2. Viscosity

The viscosity of 1% chitosan in 1% acetic acid solution was determined using a Brookfield digital rheometer (Model DV-II, Brook Engineering Laboratories, Inc., Stoughton, MA, USA) at 25 °C, Spindle CPE-40, 0.5 mL sample volume.

#### 3.8.3. Molecular Weights of Chitosan

The molecular weights of chitosan were determined by viscosity, using the procedure described by Fai *et al.* [4]. The viscosity of chitosan was determined using an AVS-350 viscometer (Schott-Geräte, Mainz, German), type/capillary: Cannon-Fenske d_inside_ = 1.01 mm, at 25 °C. After obtaining the intrinsic viscosity from tables, K and a, were obtained for HAc/NaAc. K = 0.076, a = 0.76. The flow time was determined in seconds. Using the Mark-Houwinks equation, the average viscosimetric molecular weight was expressed in g/mol.

#### 3.8.4. Thermal Analysis

Thermogravimetric Analysis (TGA) and Differential Scanning Calorimetry (DSC) were carried out using Shimadzu model 50WS and Shimadzu model DSC-50WS thermal analysis instruments, respectively. An accurately weighed (10 mg) chitosan sample was placed in an aluminum cup and sealed. The experiment consisted of heating the samples from 0 to 400 °C under the continuous flow of dry nitrogen gas (50 mL·min^−1^), at a heating rate of 10 °C·min^−1^.

#### 3.8.5. Elemental Analysis

The elemental analysis was carried out on the elemental analyzer model EA 1110 Carlo Erba Instruments equipment and approximately 3 mg of chitin and chitosan was used. The elemental analysis was also used to determine the percentage of carbon, hydrogen and nitrogen.

#### 3.8.6. Scanning Electron Microscopy

The dried sample was ground under vacuum using a sputter coater and its surface was observed in a scanning electron microscope, Series XL 30 (Umax) Environmental Scanning Electron Microscopy (ESEM) equipped with a tungsten filament using a 20 kV accelerating voltage.

#### 3.8.7. Crystallinity Index

The X-ray diffractograms of chitin and chitosan were obtained in the X-Ray Laboratory of the Physics Department—Federal University of Pernambuco—UFPE. The measurement was taken using Siemens Model 5000 D X-ray equipment, Cu Kα radiation with λ = 1.542 Å, in a scanning range between 4° and 50° with a rate of 0.02 min^−1^. The interplanar distance was determined by the width of the half peak height of greatest intensity (IC). The crystallinity index (ICR) was determined using the following equation:

Crystallinity index (%) = 100{[I(θc) − I(θa)] / I(θc)}
(2)

where I (θc) is the relative intensity of the crystalline (2θ = 20°) and I (θa) corresponds to amorphous regions (2θ = 12°) for chitosan.

### 3.9. Cytotoxicity Test using Chorioallantoic Membrane (HET-CAM Test)

To evaluate the cytotoxicity and biocompatibility of chitin and chitosan, Hen's Egg Tests (HETs) were performed on the chorioallantoic membrane (HET-CAM) as per the methodology described by Steiling *et al.* [47]. All assays were repeated five times. Membranes were observed for 5 min for signs of vasoconstriction, haemorrhage and coagulation. The time (in seconds) at which the indicated processes began were applied in Equation (1) [50]:

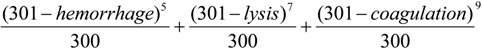
(3)

After application of the formula above, it was possible to quantify the observed potential for irritation (irritation score-IS) and to obtain means and standard deviations for the analysis as follows: 0–0.9 no irritation, 1–4.9 slight irritation, 5–8.9 moderate irritation and 9–21 severe irritation [50].

## 4. Conclusions

Considering the results obtained with corn steep liquor (CSL) and cassava wastewater (CW) these renewable substrates may be used as alternative substrate sources for chitin and chitosan production by *Cunninghamella elegans*. The use of tropical residues as nutrients for the large-scale production of these biopolymers is viable, economic and environmentally friendly. The microbiological chitin and chitosan obtained are not irritating, are biocompatible and have chemical properties that enable it to be used for biotechnological applications. The fundamental data obtained contributes to our understanding of the abilities and potential of *C. elegans*, and effectively reduced the costs of chitin and chitosan production.

## References

[1] Dhillon G.S., Kaurc S., Sarma S.J., Brar S.K. (2013). Integrated process for fungal citric acid fermentation using apple processing wastes and sequential extraction of chitosan from waste stream. Ind. Crop. Prod..

[2] Stamford T.C.M., Stamford T.L.M., Stamford N.P., Neto B.B., Campos-Takaki G.M. (2007). Growth of *Cunninghamella. elegans* UCP 542 and production of chitin and chitosan using yam bean medium. Electron. J. Biotechnol..

[3] Bueter C.L., Specht C.A., Levitz S.M. (2013). Innate Sensing of Chitin and Chitosan. PLoS Pathog..

[4] Fai A.E.C., Stamford TC.M., Stamford-Arnaud T.M., Santa-Cruz P.A., Silva M.C.F., Campos-Takaki G.M., Stamford T.L.M. (2011). Physico-chemical characteristics and functional properties of chitin and chitosan produced by *Mucor. circinelloides* using yam bean as substrate. Molecules.

[5] Arbia W., Adour L., Amrane A., Lounici H. (2013). Optimization of medium composition for enhanced chitin extraction from *Parapenaeus. longirostris* by *Lactobacillus helveticus* using response surface methodology. Food Hydrocolloids.

[6] Berger L.R.R., Cardoso A., Stamford T.C.M., Cavalcante H.M.M., Macedo R.O., Campos-Takaki G.M. (2011). Agroindustrial waste as alternative medium in the production of chitin and chitosan by *Rhizopus. arrhizus*—A factorial design. Asian Chitin J..

[7] Cardoso A., Silva M.C.F., Batista A.C., Campos-Takaki G.M. (2010). Submerged fermentation for chitin and chitosan production by *Rhizopus. arrhizus* UCP 402. Asian Chitin J..

[8] Stamford T.C.M., Stamford-Arnaud T.M., Cavalcante H.M.M., Macedo R.O., Campos-Takaki G.M., Andrade A.O., Pereira A.A., Naves E.L.M., Soares A.B. (2013). Microbiological chitosan: Potential application as anticariogenic agent. Practical Applications in Biomedical Engineering.

[9] Carvalho M.M.S.G., Stamford T.C.M., Santos E.P., Tenorio P., Sampaio F. (2012). Chitosan as an oral antimicrobial agent. Science against Microbial Pathogens: Communicating Current Research and Technological Advances.

[10] Pochanavanich P., Suntornsuk W. (2002). Fungal chitosan production and its characterization. Lett. Appl. Microbiol..

[11] Mario F.D., Rapana P., Tomati U., Galli E. (2008). Chitin and chitosan from Basidiomycetes. Int. J. Biol. Macromol..

[12] Tan S.C., Tan T.K., Sek M.W., Khorb E. (1996). The chitosan yield of zygomycetes at their optimum harvesting time. Carbohydr. Polym..

[13] Tajdini F., Amini M.A., Nafissi-Varcheh N., Faramarzi M.A. (2010). Production, physiochemical and antimicrobial properties of fungal chitosan from *Rhizomucor miehei* and *Mucor racemosus*. Int. J. Biol. Macromol..

[14] nwe N., Stevens W.F. (2004). Effect of urea on fungal chitosan production in solid substrate fermentation. Process. Biochem..

[15] Batista A.C.L., Silva M.C.F., Batista J.B., Nascimento A.E., Campos-Takaki G.M. (2013). Eco-friendly chitosan production by *Syncephalastrum racemosum* and application to the removal of acid orange 7 (AO7) from wastewaters. Molecules.

[16] Santos E.R., Silva M.C.F., Souza P.M., Silva A.C., Paiva S.C., Albuquerque C.D.C., Nascimento A.E., Okada K., Campos-Takaki G.M. (2013). Enhancement of *Cunninghamella elegans* UCP/WFCC 0542 biomass and chitosan with amino acid supply. Molecules.

[17] Hamano P.S., Kilikian B.V. (2006). Production of red pigments by *Monascus. ruber* in culture media containing corn steep liquor. Braz. J. Chem. Eng..

[18] Karimi K., Zamani A. (2013). *Mucor indicus*: Biology and industrial application perspectives: A review. Biotechnol. Adv..

[19] Lins C.I.M., Cardoso A., Silva M.C.F., Batista A.C.L., Jara A.M.A.T., Berger L.R.R., Santos E.R., Marques Da Silva A., Campos-Takaki G.M. (2010). Evaluation of chitin and chitosan by different extraction methods from mucoralean fungi biomass. Asian Chitin J..

[20] Cardoso A., Lins C.I.M., Santos E.R., Silva M.C.F., Campos-Takaki G.M. (2012). Microbial enhance of chitosan production by *Rhizopus arrhizus* using agroindustrial substrates. Molecules.

[21] Xi Y., Chen K., Dai W., Ma J., Zhang M., Jiang M., Wei P., Ouyang P. (2013). Succinic acid production by *Actinobacillus succinogenes* NJ113 using corn steep liquor (CSL) powder as nitrogen source. Bioresource. Technol..

[22] Sobrinho H.B.S., Luna J.M., Rufino R.D., Porto A.L.F., Sarubbo L.A. (2013). Assessment of toxicity of a biosurfactant from *Candida sphaerica* UCP 0995 cultivated with industrial residues in a bioreactor. Electron. J. Biotechnol..

[23] New N., Chandrkrachang, Stevens W.F., Maw T., Tan T.K., Khor E., Wong S.M. (2002). Production of fungal chitosan by solid state and submerged fermentation. Carbohydr. Polym..

[24] White S.A., Farina P.R., Fultong L. (1979). Production and isolation of chitosan from *Mucor rouxii*. Appl. Environ. Microbiol..

[25] Amorin R.V.S., Souza W., Fukushima K., Campos-Takaki G.M. (2001). Faster chitosan production by mucoralean strains in submerged culture. Braz. J. Microbiol..

[26] Chatterjee S., Adhya M., Guha A.K., Chatterjee B.P. (2005). Chitosan from *Mucor. rouxii*: Production and physico-chemical characterization. Process. Biochem..

[27] Ebrahimzadeh M.A., Chabra A., Gharaei-Fathabad E., Pourmorad F. (2013). Preparation of chitosan from *Penicillium.* spp. and determination of their degree of deacetylation. Indian J. Biotechnol..

[28] Santos J.E., Soares J.P., Dockal E.R., Campana Filho S.P., Cavalheiro E.T.G. (2003). Caracterização de quitosanas comerciais de diferentes origens. Polimeros.

[29] Khalaf S.A. (2004). Production and characterization of fungal chitosan under solid-state fermentation conditions. Int. J. Agric. Biol..

[30] Omogbai B.A., Ikenebomeh M. (2013). Solid-state fermentative production and bioactivity of fungal chitosan. J. Microbiol. Biotechnol. Food Sci..

[31] Tikhonov V.E., Stepnova E.A., Babak V.G., Yamskov I.A., Palma-Guerrero J., Jansson H.B., Lopez-Llorca L.V., Salinas J., Gerasimenko D.V., Avdienko I.D. (2006). Bactericidal and antifungal activities of a low molecular weight chitosan and its N-/2(3)-(dodec-2-enyl)succinoyl/-derivatives. Carbohydr. Polym..

[32] Rong H.C., Horng-Dar H. (1996). Effect of molecular weight of chitosan with the same degree of deacetylation on the thermal, mechanical, and permeability properties of the prepared membrane. Carbohydr. Polym..

[33] Cho Y.I., No H.K., Meyers S.P. (1998). Physicochemical characteristics and functional properties of various commercial chitin and chitosan products. J. Agric. Food. Chem..

[34] Ming-Tsung Y., Joan-Hwa Y., Jeng-Leun M. (2009). Physicochemical characterization of chitin and chitosan from crab shells. Carbohydr. Polym..

[35] Sreenivasan K. (1996). Thermal stability studies of some chitosan-metal ion complexes using differential scanning calorimetry. Polym. Degrad. Stabil..

[36] Kittur F.S., Prashanth K.V.H., Sankar K.V., Tharanathan R.N. (2002). Characterization of chitin, chitosan and their carboxymethyl derivatives by differential scanning calorimetry. Carbohydr. Polym..

[37] Zhang H., Yanga S., Fanga J., Denga Y., Wanga D., Zhao Y. (2014). Optimization of the fermentation conditions of Rhizopus japonicusM193 for the production of chitin deacetylase and chitosan. Carbohydr. Polym..

[38] Ming-Tsung Y., Jeng-Leun M. (2007). Physico-chemical characterization of fungal chitosan from shiitake stipes. LWT Food Sci. Technol..

[39] Zawadzki J., Kaczmarek H. (2010). Thermal treatment of chitosan in various conditions. Carbohydr. Polym..

[40] Liu H., Du Y., Yang J., Zhu H. (2004). Structural characterization and antimicrobial activity of chitosan/betaine derivative complex. Carbohydr. Polym..

[41] Chan H.-Y., Chen M.-H., Yuan G.-F. (2001). Fungal chitosan. Fungal Sci..

[42] Wang W., Du Y., Qiu Y., Wang X., Hu Y., Yang J., Cai J., Kennedy J.F. (2008). A new green technology for direct production of low molecular weight chitosan. Carbohydr. Polym..

[43] Pareek N., Vivekanand V., Agarwal P., Saroj S., Singh R.P. (2013). Bioconversion to chitosan: A two stage process employing chitin deacetylase from Penicillium oxalicum SAEM-51. Carbohydr. Polym..

[44] Bagley D.M., Waters D., Kong B.M. (1994). Development of a 10-day chorioallantoic membrane vascular assay as an alternative to the Draize rabbit eye irritation test. Food Chem. Toxicol..

[45] Vargas A., Zeisser-Labouèbe M., Lange N., Gurny R., Delie F. (2007). The chick embryo and its chorioallantoic membrane (CAM) for the *in vivo* evaluation of drug delivery systems. Adv. Drug Deliv. Rev..

[46] Luepke N. (1985). Hen’s egg chorioallantoic membrane test for irritation potential. Food Chem. Toxic.

[47] Steiling W., Bracher M., Coutellemont P., Silva O. (1999). The HET-CAM, a useful *in vitro* assay for assessing theeye irritation properties of cosmetic formulations and ingredients. Toxicol. In Vitro.

[48] Jin Hu K., Yeung K.W., Ho K.P., Hu J.L. (1999). Rapid extraction of high-quality chitosan from mycelia of *Absidia glauca*. J. Food Biochem..

[49] Baxter A., Dillon M., Taylor K.D.A., Roberts G.A.F. (1992). Improved method for IR determination of the degree of N-acetylation of chitosan. Int. J. Biol. Macromol..

[50] Kalweit S., Besoke R., Gerner I., Spielmann H. (1990). A national validation project of alternative methods to the Draize rabbit eye test. Toxicol. In Vitro.

